# Cardiological Disorders Leading to Ineligibility for Compulsory Military Service

**DOI:** 10.3390/medicina62050802

**Published:** 2026-04-22

**Authors:** Tautvydas Ribinskas, Tautvydas Rugelis, Eglė Labanauskaitė, Vilius Kviesulaitis, Vytautas Zabiela, Tomas Kazakevičius

**Affiliations:** 1 Faculty of Medicine, Medical Academy, Lithuanian University of Health Sciences, 44307 Kaunas, Lithuania; 2 Heart Centre, Kauno Klinikos, Hospital of Lithuanian University of Health Sciences, 50161 Kaunas, Lithuania; 3 Heart Centre, Medical Academy, Lithuanian University of Health Sciences, 44307 Kaunas, Lithuania; 4 The Institute of Cardiology, Lithuanian University of Health Sciences, 50103 Kaunas, Lithuania; 5 Kaunas Regional Society of Cardiologists, 50009 Kaunas, Lithuania

**Keywords:** cardiovascular diseases, military service eligibility, conscription, hypertension, rhythm disorders, cardiac eligibility

## Abstract

*Background and Objectives*: Cardiovascular disorders contribute substantially to medical ineligibility for compulsory military service in Lithuania. This study aimed to describe cardiovascular disease patterns and assess their association with military service eligibility among conscription-age individuals treated at a tertiary care center, considering gender, place of residence, age, and the most common cardiovascular causes of medical ineligibility. *Materials and Methods*: This retrospective hospital-based study included men and women aged 18–26 years with cardiovascular disease diagnoses defined according to ICD-10 codes specified by the Order of the Minister of National Defense of the Republic of Lithuania. Anonymized medical records from the Hospital of the Lithuanian University of Health Sciences Kaunas Clinics were reviewed. Participants were categorized into four military service eligibility classes based on medical history data and were stratified by gender, age, and place of residence. *Results*: The study included 521 participants (56.6% male, 43.4% female). Gender and residence showed no significant impact on military service eligibility. Younger individuals, particularly those aged 18–19, were more often deemed eligible, while eligibility declined with age. Males more commonly had essential hypertension and hypertensive heart disease, whereas females more frequently presented with paroxysmal tachycardia and other arrhythmias. Hypertension and other severe cardiovascular conditions most strongly reduced eligibility for compulsory military service, whereas rhythm disorders were more often compatible with service. *Conclusions*: In this hospital-based cohort of conscription-age individuals with cardiovascular disease, gender and place of residence did not significantly influence eligibility for military service. Eligibility declined with increasing age, and hypertension-related cardiovascular disorders were the leading cause of ineligibility among conscripts.

## 1. Introduction

On 11 March 1990, Lithuania restored its statehood and re-established itself as the Republic of Lithuania, making the reconstruction of defense institutions a state priority. Between 1991 and 1993, the Lithuanian Armed Forces were reconstituted [1]. In response to increasing geopolitical challenges, in February 2015, the Seimas of the Republic of Lithuania reintroduced compulsory military service, which had been abolished in 2008. It was decided that each year 3500–4500 young males aged 18 to 23 would be randomly called up for nine months of mandatory military service [2].

Under the consolidated law, conscripts are selected from the unprepared personnel reserve, primarily between 18 and 23 years of age, with voluntary service permitted up to 38 years. Individuals whose service has been deferred due to public duties are required to complete conscription by age 26 once the deferral conditions are no longer applicable. In Lithuania, only men are conscripted for compulsory military service, while women may serve only if they volunteer [3]. However, considering growing geopolitical challenges and threats to Lithuania’s independence, political discussions have increasingly focused on the possibility of introducing universal conscription, with both men and women being called into the service, similar to the model already implemented in Norway [4,5].

In 2002, by resolution of the Government of the Republic of Lithuania, the Regulations on Military Medical Expertise were adopted. These regulations govern the medical examination of Lithuanian military personnel, during which their fitness for the military service is assessed [6]. This order specifies the methodology according to which the fitness of service members is evaluated [7].

According to the Lithuanian State Audit, over half of the conscripts evaluated by the military medical commission were deemed unfit for the service: 51% in 2022, 54% in 2023, and 51% in 2024. In 2024, mental and psychological disorders were the primary cause of unfitness, while somatic diseases, including cardiovascular conditions, accounted for 23% of the cases, representing a significant proportion of medical disqualifications [8]. While legal and procedural frameworks ensure systematic medical evaluation, the prevalence of cardiovascular diseases (CVDs) among young conscripts remains a critical concern due to its implications for service eligibility.

Although the prevalence of CVD in the middle-aged Lithuanian population has been decreasing, Lithuania ranks amongst the worst of European countries. According to the 2023 European Observatory report, nearly half of all deaths (48.7%) in Lithuania were attributed to CVD [9,10]. Data from the Lithuanian Institute of Hygiene indicates that, in 2024, CVD were diagnosed in 13,193 young individuals aged 18–26 years. The prevalence of CVD in the general population is relatively low (approximately 1.3%) at conscription age, yet these conditions are highly significant due to their impact on fitness and eligibility for military service [11].

A study by Beijian Zhang and colleagues, examining individuals aged 15–44, reported a 45.5% increase in the prevalence of CVD over 30 years, with the rise more pronounced in regions of lower socioeconomic status. Key risk factors included high systolic blood pressure, elevated low-density lipoprotein, high body mass index, air pollution, and smoking [12]. Similar risk factors affect citizen conscripts: A 2020 study found that nearly half were physically inactive, had unhealthy diets, and 62.3% smoked regularly [13], increasing their risk of CVD and potential disqualification from military service. Young adult men and women differ in their cardiovascular risk profiles. Studies consistently show that men in early adulthood have higher rates of hypertension, adverse lipid levels, and unhealthy lifestyle patterns, resulting in a higher overall CVD risk. Women of the same age generally exhibit more favorable cardiovascular profiles, although obesity and lower physical activity are more common [14].

Given the high proportion of conscripts deemed unfit due to somatic conditions, it is important to identify which cardiovascular disorders significantly contribute to ineligibility for military service. Therefore, the aim of this study was to analyze the prevalence of CVD among conscription-age individuals and to assess their role as a determining factor in medical disqualification from compulsory military service.

## 2. Materials and Methods

The study included men and women aged 18–26 years with cardiovascular diagnoses who were treated at the Hospital of the Lithuanian University of Health Sciences Kaunas Clinics in 2024, forming a hospital-based cohort of conscription-age individuals. Only the patients with ICD-10 codes for CVD were included. These ICD-10 codes were selected because they are explicitly listed as cardiovascular conditions to be evaluated during military medical expertise for conscription eligibility in the regulations issued by the Minister of National Defense of the Republic of Lithuania. The codes referred were I00–I09, I10–I15, I20–I25, I26–I28, I30–I52, I70, I71, I72, I73, I95–I99, and R00.0–R00.1. Anonymized medical records from the Hospital of the Lithuanian University of Health Sciences Kaunas Clinics were reviewed. ICD-10 codes with 10 or fewer cases each were grouped into an “Other” category. Individual ICD-10 subcodes were aggregated into broader parent categories to ensure adequate group sizes for statistical analyses. When multiple cardiovascular ICD-10 codes were recorded for the same individual, all codes were retained for descriptive summaries, whereas for regression analyses, each participant was assigned to the diagnostic group judged to have the greatest impact on military eligibility according to the regulatory criteria.

Based on the official methodology for military medical examinations and the available medical history data, participants were categorized into four classes reflecting their presumed eligibility for military service. In these regulations, specific cardiovascular conditions and their severity are explicitly linked to defined eligibility categories, specifying which conditions correspond to each eligibility class for military service. This reconstructed classification served as a proxy for formal military medical commission decisions, as it was derived solely from medical records and did not constitute an official military medical examination.

Class 1—Eligible for compulsory initial military service, active reserve, Lithuanian Riflemen’s Union combat units, professional military service, or military education institutions.

Class 2—Eligible for international operations.

Class 3—Eligible for further professional military service, including service in intelligence institutions under contract; graduates of military education institutions entering professional service; training in foreign military institutions; reservists previously serving in professional military service re-entering professional service or active reserve; or further service in the Lithuanian Riflemen’s Union combat units, the prepared reserve, or reserve units mobilized for training.

Class 4—Eligible for compulsory military service during mobilization and for soldiers serving under mobilization [7].

Eligibility proportions vary within the ICD-10 parent codes because these categories group multiple subcodes with different clinical implications. Beyond aggregation into ICD-10 parent categories, diagnostic groups were further consolidated into clinically meaningful clusters for regression analyses (Hypertension, Arrhythmia, Valvular, Conduction), and all remaining cardiovascular diagnoses were classified as “Other”. Logistic regression models were therefore restricted to these aggregated groups, while models for rarer patterns were not interpreted due to unstable estimates and non-informative confidence intervals. Logistic regression was used to estimate odds ratios and 95% confidence intervals for ineligibility for military service, adjusting for age, gender, place of residence, and ICD-10 based diagnostic group. Participants were stratified according to place of residence: urban areas (>3000 inhabitants) and rural areas (<3000 inhabitants). Statistical analysis was performed using IBM SPSS Statistics version 30.0. The *χ*^2^ test was used to assess statistical significance, and Monte Carlo simulation (10,000 samples) was applied in cases of small expected frequencies. Ethical approval for the study was obtained from the Bioethics Center of LUHS (2025-BEC2-0506).

## 3. Results

The study included 521 participants, of whom 295 were male (56.6%) and 226 were female (43.4%). A total of 378 participants resided in urban areas (72.6%), while 143 lived in non-urban areas (27.4%). The largest group of participants was the age of 23 years old (*n* = 74, 14.2%), while the smallest at 21 years old (*n* = 44, 8.4%).

The analysis revealed no statistically significant differences between the male and female participants in terms of eligibility for the various classes of military service (Table 1). A near-significant trend was observed in Class 4. These findings suggest that gender is not a major determinant of eligibility for military service among the individuals diagnosed with CVD.

Age was significantly associated with eligibility for military service across all classes, with overall eligibility decreasing as the age increased (Table 2). In Class 1, individuals aged 18–19 were the most frequently deemed eligible for service. In Class 2, a similar pattern was observed for the eligibility for international operations: 18- and 19-year-olds were more often eligible than expected, while 25-year-olds were disproportionately classified as ineligible. The 21-year-old group more frequently required individual assessment, indicating the need for additional medical evaluation before the final eligibility determination. Class 3 showed a significant relationship between the age and eligibility for the professional military service, including contract service in intelligence institutions, graduates of military education institutions entering professional roles, training in foreign military schools, reservists returning to active duty, and further service in the Lithuanian Riflemen’s Union units or mobilized reserves, with 24-year-olds disproportionately classified as ineligible. In Class 4, corresponding to the mobilization group, individuals across all age groups were more frequently deemed eligible for service.

Comparison between the urban and rural participants revealed no statistically significant differences in eligibility across the four classes (Table 3). Minor variations were observed: urban residents exhibited slightly higher eligibility in Classes 1 and 2, whereas rural residents were more frequently classified as ineligible in Classes 2 and 4; however, these differences were not statistically significant. Proportions of participants requiring individual assessment in Class 2 were comparable between the two groups.

The distribution of ICD-10 diagnostic categories differed significantly between males and females (Table 4). Among males, essential hypertension and hypertensive heart disease occurred more frequently, whereas these diagnoses were less frequent in females. Conversely, paroxysmal tachycardia and other cardiac arrhythmias were more common in females than in males. By place of residence, no significant differences were observed.

The distribution of military service eligibility differed significantly across ICD-10 diagnostic categories in all four classes (Table 5).

In Class 1, participants with hypertensive heart disease and conditions classified as “Other” were deemed ineligible more often than expected based on the χ^2^ residuals, whereas participants with paroxysmal tachycardia and other cardiac arrhythmias were more frequently classified as eligible than expected. Essential hypertension showed a higher-than-expected rate of ineligibility. The “Other” category comprised rare but severe conditions, such as aortic aneurysm and aortic coarctation, which are generally incompatible with military service.

In Class 2, hypertensive heart disease and pulmonary embolism were associated with higher-than-expected rates of ineligibility, while paroxysmal tachycardia and other arrhythmias were more often classified as eligible.

In Class 3, essential hypertension and hypertensive heart disease were again more frequently deemed ineligible than expected, whereas paroxysmal tachycardia and other arrhythmias showed higher-than-expected eligibility.

In Class 4, ineligibility was disproportionately high for hypertensive heart disease and “Other” conditions, while paroxysmal tachycardia and other arrhythmias were more commonly classified as eligible than expected.

Overall, hypertension and “Other” severe cardiovascular conditions markedly reduced eligibility for compulsory military service, whereas paroxysmal tachycardia and rhythm disorders were associated with higher eligibility.

In Class 4, unsuitability was disproportionately high for hypertensive heart disease and “Other” conditions, while paroxysmal tachycardia and other arrhythmias were more commonly classified as suitable than expected.

Overall, hypertension and “Other” severe cardiovascular conditions markedly reduced suitability for compulsory military service, whereas paroxysmal tachycardia and rhythm disorders were associated with higher eligibility.

In multivariable analysis, the pattern of associations between major cardiovascular diagnoses and medical ineligibility was largely consistent across all four eligibility classes (Table 6). Compared with hypertension, arrhythmias were associated with substantially lower odds of disqualification in every class (adjusted OR 0.25 in Class 1, 0.48 in Class 2, and 0.12 in Classes 3 and 4; all *p* < 0.010), while valvular disease also showed reduced odds across classes (adjusted OR 0.40, 0.50, 0.28, and 0.29, respectively). In contrast, the heterogeneous group of “Other” CVD demonstrated the strongest adverse association with eligibility, particularly in Classes 1, 2, and 4 (OR 3.04, 4.75, and 1.96, respectively), reflecting the inclusion of less common cardiovascular conditions with variable clinical implications.

Age acted as an additional determinant mainly in the stricter Classes 1 and 2, where each year of increase was associated with a gradual rise in the probability of ineligibility (18% and 21% increase per year, respectively), whereas in Classes 3 and 4, age no longer had a significant impact. Gender had a measurable impact on eligibility only in Class 1, where males were 41% less likely to be deemed ineligible than females with a statistically significant association, while in Classes 2–4, no independent gender-related differences in eligibility were observed.

## 4. Discussion

This study examined the impact of cardiovascular disorders on eligibility for military service among conscription-age individuals. The findings indicated that gender did not have a statistically significant effect on service eligibility with respect to CVD, suggesting that men and women have comparable medical eligibility. Similarly, a 2022 study from Norway reported that increased female participation in compulsory military service did not reduce overall physical readiness—particularly cardiorespiratory endurance—and may even have contributed to improvements [15]. These findings indicate that gender differences do not inherently limit the physical or operational capacity of military units. In many NATO allies armed forces, women now constitute an increasing share of both conscripted and professional personnel [16], and medical eligibility criteria are increasingly being defined in a transparent and sex-neutral manner. In Lithuania, compulsory military service currently applies only to men, with women serving on a voluntary basis, although the possibility of extending conscription to women has been increasingly discussed at the political level. In this context, the absence of marked sex-related differences in cardiovascular eligibility in our hospital-based cohort indicates that within the limits of this sample, we did not identify major cardiological barriers to potential future policies that would expand conscription to include women in Lithuania.

Regardless of gender, demographic trends also have important implications for the future pool of medically eligible conscripts. Lithuania is facing an aging population, reflected by an increasing median age and a positive demographic aging coefficient [17]. This study found that eligibility for military service declines with age, with individuals aged 18–19 being the most eligible. Consequently, the number of individuals medically eligible for service is decreasing in an aging society. In response, a 2025 Lithuanian conscription reform will introduce health checks for 17-year-olds, who will become eligible for service at 18 [3]. These measures aim to address the impact of demographic changes on the pool of eligible conscripts.

This study also examined the impact of place of residence. Previous research has shown that living in sparsely populated areas may reduce the risk of hypertension [18], yet access to healthcare in rural regions has declined due to a steady decrease in the number of physicians since 1990 [19]. Despite these disparities, no significant differences in CVD were found between urban and rural conscription-age individuals, suggesting that residence has a limited impact on cardiovascular health within this age group. In this narrowly defined, generally healthy age group, lifestyle patterns and access to urgent or specialist care may be relatively similar across regions, which could partly explain the absence of major rural–urban differences in our cohort despite long-term reductions in physician density in rural areas. However, if the decline in physician availability in rural areas continues, it may further compromise the early diagnosis and treatment of cardiovascular conditions, and future cohorts of conscription-age individuals might begin to exhibit clearer rural–urban disparities in cardiovascular risk profiles and medical eligibility for service.

In contrast, this study identified clear sex-related differences in CVD distribution: hypertensive disorders were more common in males, while cardiac arrhythmias were more prevalent in females. Previous research confirms an increasing trend of hypertension among men and a decline among women over the past four decades [20]. Although overall arrhythmia prevalence is not strongly gender-specific, ventricular arrhythmias are more common in men and supraventricular arrythmias in women [21]. From a practical perspective, these findings highlight the importance of systematic blood pressure screening and lifestyle counselling in all conscription-age individuals, with particular attention to young men, who tend to have a higher burden of hypertension and traditional cardiovascular risk factors, and to careful, diagnosis-specific assessment of arrhythmias in both genders rather than treating all rhythm disorders as uniform contraindications to military service.

In the assessment of conscript’s eligibility for compulsory military service, hypertensive heart disease was identified as the main cause of disqualification. Hypertension in young individuals is particularly concerning, as it often remains asymptomatic and undetected, especially during the transition from pediatric to adult healthcare, when early diagnosis and management are frequently delayed. Consequently, many cases are discovered only after complications such as myocardial dysfunction, arrhythmias, or acute myocardial infarction have developed [22,23]. The physical and psychological stress associated with military service can further accelerate these processes. Moreover, early-onset hypertension is linked to endothelial dysfunction, increased arterial stiffness, and left ventricular hypertrophy, which reduce cardiovascular reserve and elevate the risk of acute cardiovascular events during service [24]. These pathophysiological alterations not only impair physical performance but also increase the risk of acute cardiovascular events during military service. In our cohort, these adverse effects were most evident among individuals with hypertensive heart disease (I11), who were consistently classified as ineligible for service more often than those with essential hypertension (I10). This difference likely reflects the cumulative impact of long-standing or poorly controlled hypertension leading to structural cardiac damage, which is explicitly recognized in the military medical regulations by assigning stricter eligibility criteria to parent codes that indicate target-organ involvement.

Cardiac arrhythmias were more frequently associated with eligibility for service than expected in this cohort. This likely reflects the broad definition of arrhythmias in our sample, which encompassed several relatively common and non-life-threatening rhythm disturbances, such as sinus tachycardia, frequent extrasystoles, and paroxysmal supraventricular tachycardias. As a result, the presence of an arrhythmia diagnosis did not necessarily lead to disqualification from compulsory military service, particularly when these conditions were benign and not accompanied by structural heart disease or significant symptoms.

Overall, these findings underscore the critical role of cardiovascular health in determining eligibility for compulsory military service and highlight the importance of the early detection and management of hypertension and other modifiable cardiovascular risk factors, which may help maintain or increase the pool of medically eligible conscripts and support long-term military readiness.

## 5. Conclusions

Eligibility for compulsory military service was not significantly affected by gender or residential location in this hospital-based cohort of conscription-age individuals with cardiovascular disease. Age was a significant determinant, with younger individuals (18–19 years) more likely to be deemed eligible than older conscripts. Hypertension and hypertensive heart disease strongly predicted medical ineligibility, whereas many arrhythmias and other heart rhythm disorders were more often compatible with service. These findings highlight the importance of targeted medical screening for hypertension and other modifiable risk factors while recognizing that not all CVDs equally compromise service potential and suggest that strengthening early cardiovascular prevention in adolescence and young adulthood may help improve population health and support future defense readiness.

### Limitations of the Study

This study has several limitations that should be considered when interpreting the findings. The retrospective, single-center design based on medical records from a tertiary care hospital may limit the generalizability of the findings, as only conscription-age individuals treated at this institution were included, whereas potential conscripts managed in other healthcare settings were not captured. In addition, LUHS Kaunas Clinics is the largest tertiary care hospital in Lithuania and serves as a referral center for more complex cardiovascular cases. Young adults with CVD may therefore be preferentially referred to this setting, potentially shifting the case mix toward more severe disease and influencing the distribution of diagnoses and eligibility outcomes observed in this study. However, such referral-related differences were not formally assessed.

The categorization into military service eligibility classes was reconstructed based on clinical documentation and the official military medical expertise methodology; however, these reconstructed classifications were not directly validated against the actual decisions of military medical commissions. As a result, some cases—particularly borderline conditions requiring individualized assessment—may have been misclassified. The “Other” CVD category comprised heterogeneous and relatively rare conditions, precluding more granular analysis of individual diagnoses and potentially diluting or exaggerating their specific effects. In addition, the assignment of participants to reconstructed eligibility classes was performed by a single investigator, which may have introduced observer bias, although standardized criteria were applied consistently across all cases.

Furthermore, several potentially relevant variables, such as physical eligibility test results, lifestyle factors (including smoking, physical activity, and diet), psychosocial stress, and treatment adherence, were not systematically available in the medical records and therefore could not be included in the multivariable models, leaving the possibility of residual confounding. Despite these limitations, the use of a relatively large and well-defined cohort of conscription-age individuals from a tertiary care center provides a valuable overview of how specific cardiovascular diagnoses influence medical eligibility for military service.

## Figures and Tables

**Table 1:** Distribution of participants by gender and suitability for different military service classes.

	Class 1		Class 2			Class 3		Class 4	
	Ineligible *n* (%)	Eligible *n* (%)	Ineligible *n* (%)	Eligible *n* (%)	Individually *n* (%)	Ineligible *n* (%)	Eligible *n* (%)	Ineligible *n* (%)	Eligible *n* (%)
Male	176 (59.7)	119 (40.3)	119 (40.3)	119 (40.3)	57 (19.3)	52 (17.6)	243 (82.4)	64 (21.7)	231 (78.3)
Female	140 (61.9)	86 (38.1)	99 (43.8)	86 (38.1)	41 (18.1)	31 (13.7)	195 (86.2)	34 (15.0)	192 (85.0)
Total	316 (60.7)	205 (39.3)	218 (41.8)	205 (39.3)	98 (18.8)	83 (15.9)	438 (84.1)	98 (18.8)	423 (81.2)
	*χ*^2^ (df = 1) = 0.280; *p* = 0.597		*χ*^2^ (df = 2) = 0.632; *p* = 0.729			*χ*^2^ (df = 1) = 1.461; *p* = 0.227		*χ*^2^ (df = 1) = 3.706; *p* = 0.054	

Abbreviations: *n*, number of participants.

**Table 2:** Distribution of participants by age and suitability for different military service classes.

	Class 1		Class 2			Class 3		Class 4	
Age	Ineligible *n* (%)	Eligible *n* (%)	Ineligible *n* (%)	Eligible *n* (%)	Individually *n* (%)	Ineligible *n* (%)	Eligible *n* (%)	Ineligible *n* (%)	Eligible *n* (%)
18	18 (37.5) ^a^	30 (62.5) ^b^	12 (25.0)	30 (62.5) ^b^	6 (12.5)	6 (12.5)	42 (87.5)	7 (14.6)	41 (85.4)
19	21 (38.2) ^a^	34 (61.8) ^b^	16 (29.1)	34 (61.8) ^b^	5 (9.1)	4 (7.3)	51 (92.7)	5 (9.1)	50 (90.9)
20	27 (56.3)	21 (43.8)	19 (39.6)	21 (43.8)	8 (16.7)	11 (22.9)	37 (77.1)	12 (25.0)	36 (75.0)
21	30 (68.2)	14 (31.8)	16 (36.4)	14 (31.8)	14 (31.8) ^b^	4 (9.1)	40 (90.9)	4 (9.1)	40 (90.9)
22	43 (59.7)	29 (40.3)	28 (38.9)	29 (40.3)	15 (20.8)	13 (18.1)	59 (81.9)	19 (26.4)	53 (73.6)
23	51 (68.9)	23 (31.1)	31 (41.9)	23 (31.1)	20 (27.0)	9 (12.2)	65 (87.8)	11 (14.9)	63 (85.1)
24	45 (73.8)	16 (26.2)	31 (50.8)	16 (26.2)	14 (23.0)	17 (27.9) ^b^	44 (72.1)	18 (29.5)	43 (70.5)
25	49 (74.2)	17 (25.8)	38 (57.6) ^b^	17 (25.8)	11 (16.7)	14 (21.2)	52 (78.8)	16 (24.2)	50 (75.8)
26	32 (60.4)	21 (39.6)	27 (50.9)	21 (39.6)	5 (9.4)	5 (9.4)	48 (90.6)	6 (11.3)	47 (88.7)
Total	316 (60.7)	205 (39.3)	218 (41.0)	205 (39.3)	98 (18.8)	83 (15.9)	438 (84.1)	98 (18.8)	423 (81.2)
	*χ*^2^ (df = 8) = 35.506 *p* < 0.001		*χ*^2^ (df = 16) = 47.440 *p* < 0.001			MC *χ*^2^ (df = 8) = 17.350 *p* = 0.027		*χ*^2^ (df = 8) = 19.145 *p* = 0.014	

Abbreviations: *n*, number of participants; MC, Monte Carlo simulation; ^a^, residuals < −2; ^b^, residuals > 2.

**Table 3:** Distribution of participants by place of residence and suitability for different military service classes.

	Class 1		Class 2			Class 3		Class 4	
	Ineligible *n* (%)	Eligible *n* (%)	Ineligible *n* (%)	Eligible *n* (%)	Individually *n* (%)	Ineligible *n* (%)	Eligible *n* (%)	Ineligible *n* (%)	Eligible *n* (%)
Urban	224 (59.3)	154 (40.7)	150 (39.7)	154 (40.7)	74 (19.6)	57 (15.1)	321 (84.9)	64 (16.9)	314 (83.1)
Rural	92 (64.3)	51 (35.7)	68 (47.6)	51 (35.7)	24 (16.8)	26 (18.2)	117 (81.8)	34 (23.8)	109 (76.2)
Total	316 (60.7)	205 (39.3)	218 (41.8)	205 (39.3)	98 (18.8)	83 (15.9)	438 (84.1)	98 (18.8)	423 (81.2)
	*χ*^2^ (df = 1) = 1.120; *p* = 0.290		*χ*^2^ (df = 2) = 2.646; *p* = 0.266			*χ*^2^ (df = 1) = 0.746; *p* = 0.388		*χ*^2^ (df = 1) = 3.183; *p* = 0.074	

Abbreviations: *n*, number of participants.

**Table 4:** Distribution of ICD-10 diagnostic categories by gender and place of residence.

	R00	I10	I11	I26	I34	I42	I44	I45	I47	I49	Other
Male *n* (%)	28 (9.5)	56 (19.0) ^b^	53 (18.0) ^b^	7 (2.4)	13 (18.1)	14 (4.7)	7 (2.4)	21 (7.1)	18 (6.1) ^a^	25 (8.5) ^a^	39 (13.2)
Female *n* (%)	29 (12.8)	17 (7.5) ^a^	15 (6.6) ^a^	5 (2.2)	39 (17.3)	7 (3.1)	7 (3.1)	6 (2.7)	33 (14.6) ^b^	46 (20.4) ^b^	22 (9.7)
Total *n* (%)	57 (10.9)	73 (14.0)	68 (13.1)	12 (2.3)	66 (12.7)	21 (4.0)	14 (2.7)	27 (5.2)	51 (9.8)	71 (13.6)	61 (11.7)
Urban *n* (%)	36 (9.5)	51 (13.5)	54 (14.3)	8 (2.1)	52 (13.8)	15 (4.0)	8 (2.1)	21 (5.6)	34 (9.0)	58 (15.3)	41 (10.8)
Rural *n* (%)	21 (14.7)	22 (15.4)	14 (9.8)	4 (2.8)	14 (9.8)	6 (4.2)	6 (4.2)	6 (4.2)	17 (11.9)	13 (9.1)	20 (14.0)
Total *n* (%)	57 (10.9)	73 (14.0)	68 (13.1)	12 (2.3)	66 (12.7)	21 (4.0)	14 (2.7)	27 (5.2)	51 (9.8)	71 (13.6)	61 (11.7)

Abbreviations: *n*, number of participants; MC, Monte Carlo simulation; ^a^, residuals < −2; ^b^, residuals > 2. Chi-square test for diagnostic category by sex: *χ*^2^ (df = 10) = 62.591; *p* < 0.001 and diagnostic category by place of residence: *χ*^2^ (df = 10) = 12.686; *p* = 0.242.

**Table 5:** Distribution of participants by ICD-10 diagnosis and suitability for different military service classes.

	Class 1		Class 2			Class 3		Class 4	
ICD-10	Ineligible *n* (%)	Eligible *n* (%)	Ineligible *n* (%)	Eligible *n* (%)	Individually *n* (%)	Ineligible *n* (%)	Eligible *n* (%)	Ineligible *n* (%)	Eligible *n* (%)
R00	22 (38.6) ^a^	35 (61.4) ^b^	19 (8.7)	35 (17.1) ^b^	3 (3.1) ^a^	0 (0.0)	57 (13.0)	0 (0.0)	57 (13.5)
I10	42 (57.5)	31 (42.5)	22 (10.1)	31 (15.1)	20 (20.4)	11 (13.3)	62 (14.3)	11 (11.2)	62 (14.7)
I11	57 (83.8) ^b^	11 (16.2) ^a^	29 (13.3)	11 (5.4) ^a^	28 (28.6)	20 (24.1) ^b^	48 (11.0)	20 (20.4) ^b^	48 (11.3)
I26	12 (100.0)	0 (0.0)	12 (5.5) ^b^	0 (0.0) ^a^	0 (0.0)	12 (14.5) ^b^	0 (0.0)	12 (12.2) ^b^	0 (0.0)
I34	34 (51.5)	32 (48.5)	19 (8.7)	32 (15.6)	15 (15.3)	5 (6.0)	61 (13.9)	5 (5.1) ^a^	61 (14.4)
I42	18 (85.7)	3 (14.3)	13 (6.0)	3 (1.5)	5 (5.1)	2 (2.4)	19 (4.3)	2 (2.0)	19 (4.5)
I44	11 (78.6)	3 (21.4)	10 (4.6)	3 (1.5)	1 (1.0)	7 (8.4) ^b^	7 (1.6)	7 (7.1)	7 (1.7)
I45	16 (59.3)	11 (40.7)	12 (5.5)	11 (5.4)	4 (4.1)	6 (7.2)	21 (4.8)	6 (6.1)	21 (5.0)
I47	22 (43.1)	29 (56.9) ^b^	18 (8.3)	29 (14.1) ^b^	4 (4.1)	1 (1.2) ^a^	50 (11.4)	1 (1.0) ^a^	50 (11.8)
I49	30 (42.3) ^a^	41 (57.7) ^b^	28 (12.8)	41 (20.0) ^b^	2 (2.0) ^a^	5 (6.0)	66 (15.1)	5 (5.1) ^a^	66 (15.6)
Other	52 (85.2) ^b^	9 (14.8) ^a^	36 (16.5) ^b^	9 (4.4) ^a^	16 (16.3)	14 (16.9)	47 (10.7)	29 (29.6) ^b^	32 (7.6) ^a^
Total	316 (60.7)	205 (39.3)	218 (41.8)	205 (39.3)	98 (18.9)	83 (15.9)	438 (84.1)	98 (18.8)	423 (81.2)
	MC *χ*^2^ (df = 10) = 76.827 *p* < 0.001		MC *χ*^2^ (df = 20) = 117.812 *p* < 0.001			MC *χ*^2^ (df = 10) = 114.274 *p* < 0.001		MC *χ*^2^ (df = 10) = 135.330 *p* < 0.001	

Abbreviations: *n*, number of participants; MC, Monte Carlo simulation; ^a^, residuals < −2; ^b^, residuals > 2.

**Table 6:** Adjusted odds ratios for the most frequent cardiovascular diagnoses and demographic factors across military service eligibility classes.

		Arrythmia	Conduction	Valvular	Other	Age (1 Year Increase)	Male (vs. Female)
**Class 1**	Adjusted OR	0.25	0.91	0.40	3.04	1.18	0.59
	95% Cl	0.15–0.42	0.43–1.96	0.21–0.76	1.48–6.24	1.09–1.28	0.38–0.90
	*p* value	<0.001	0.817	0.005	0.002	<0.001	0.015
**Class 2**	Adjusted OR	0.48	1.56	0.50	4.75	1.21	0.65
	95% Cl	0.27–0.84	0.69–3.56	0.24–1.04	2.21–10.21	1.11–1.31	0.41–1.03
	*p* value	<0.010	0.287	0.063	<0.001	<0.001	0.068
**Class 3**	Adjusted OR	0.12	1.70	0.28	1.50	1.05	0.81
	95% Cl	0.05–0.30	0.78–3.71	0.10–0.77	0.82–2.75	0.95–1.17	0.47–1.39
	*p* value	<0.001	0.183	0.014	0.187	0.328	0.443
**Class 4**	Adjusted OR	0.12	1.72	0.29	1.96	1.06	0.86
	95% Cl	0.05–0.30	0.79–3.74	0.10–0.79	1.05–3.65	0.95–1.17	0.50–1.48
	*p* value	<0.001	0.175	0.016	0.033	0.291	0.589

Abbreviations: OR, odds ratio; CI, confidence interval.

## Data Availability

The data supporting the findings of this study are available from the corresponding author upon reasonable request.
